# Red Blood Cell Distribution Width: A Prognostic Marker in Patients With Type B Aortic Dissection Undergoing Endovascular Aortic Repair

**DOI:** 10.3389/fcvm.2022.788476

**Published:** 2022-05-02

**Authors:** Cheng Jiang, Anbang Liu, Lei Huang, Quanjun Liu, Yuan Liu, Qingshan Geng

**Affiliations:** ^1^Department of Cardiology, Guangdong Cardiovascular Institute, Guangdong Provincial People’s Hospital, Guangdong Academy of Medical Sciences, Guangzhou, China; ^2^School of Medicine, South China University of Technology, Guangzhou, China

**Keywords:** aortic dissection, red blood cell distribution width, thoracic endovascular aortic repair, prognostic marker, in-hospital mortality

## Abstract

**Background:**

Red blood cell distribution width (RDW) is associated with cardiovascular mortality. However, the relationship between preoperative RDW and outcomes after thoracic endovascular aortic repair (TEVAR) in type B aortic dissection (TBAD) remains to be determined.

**Methods:**

We review the records of 678 patients with TBAD and treated with TEVAR in three centers. Patients were divided into two groups according to the admission RDW cut-off by receiver operating characteristic curve analysis [≤13.5% (*n* = 278) and >13.5% (*n* = 400)]. The association between RDW and long-term mortality was evaluated using Cox survival analysis. Additionally, we used general additive models (GAM) with restricted cubic splines (RCS) to explore non-linear relationships between RDW and outcomes.

**Results:**

Subjects with a high RDW had significantly higher in-hospital mortality rates (1.4 vs. 4.3%, *P* = 0.038). A total of 70 subjects died after a median follow-up period of 3.3 years. Kaplan–Meier analysis showed that subjects with an RDW >13.5% had worse survival rates than those with lower RDW values (*P* < 0.001). Multivariate Cox proportional hazard modeling revealed that an RDW >13.5% was an independent predictor of long-term mortality (adjusted HR = 2.27, *P* = 0.006). Also, we found that there was a non-linear relationship between RDW and mortality from RCS, and RDW of 13.5% might be an inflection point to distinguish the long-term mortality risk of TBAD patients.

**Conclusion:**

As an inexpensive and routinely measured parameter, RDW holds promise as a novel prognostic marker in patients with TBAD receiving TEVAR. We found that an RDW >13.5% on admission was independently associated with increased long-term mortality.

## Introduction

Type B aortic dissection (TBAD) is a fatal disease, characterized by acute and subacute forms that present with several complications including a high mortality [(1), p. 2873–926]. Thoracic endovascular aortic repair (TEVAR) is an emerging treatment approach that has rapidly gained acceptance by clinicians for treating acute and sub-acute TBAD [(2, 3); (4), p. 407–16]. Compared to open surgery and medical treatment, TEVAR is considered a superior treatment strategy with lower mortality rates (5). However, postoperative mortality is still high during short- and long-term follow-ups [(3); (6), p. 213–25]. Therefore, early identification of patients at high risk for adverse outcomes is essential for clinicians to make the necessary therapeutic adjustments in a timely manner to improve prognosis.

Red blood cell distribution width (RDW) is a routine laboratory test parameter that reflects variations in the volume of red blood cells (RBC) volume [(7), p. 71–4]. In TBAD patients, blood entering the intima-media space may lead to blood loss. Liu et al. found that RDW was significantly higher in patients with anemia than in those with normal RBC levels [(8), p. 205–9]. In addition, RDW can be influenced by imbalanced physiological conditions, including oxidative stress, tissue hypoxia, neurohumoral hyperactivity, endothelial dysfunction, and chronic inflammation, all of which play an important role in aortic dissection (AD) [(9), p. 72–3]. In several clinical conditions, a high RDW has been associated with adverse outcomes. Therefore, we hypothesized that RDW could be a prognostic marker for patients with TBAD receiving TEVAR. The present study aimed to determine the relationship between RDW and adverse outcomes in patients with TBAD after TEVAR.

## Materials and Methods

### Patient Selection

**FIGURE 1:**
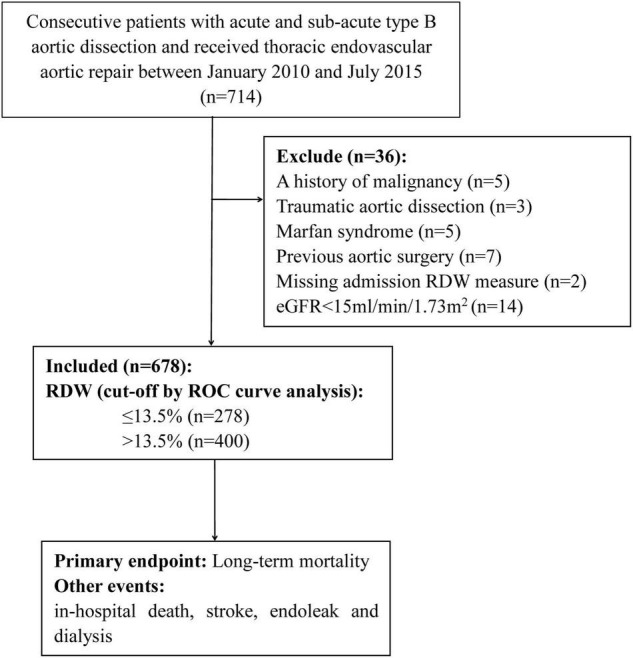
Study population flow chart.

Included in the study, were patients with consecutive TBAD who underwent TEVAR between January 2010 and July 2015, they were retrospectively identified at the Guangdong Provincial People’s Hospital (Guangzhou, China), Nanhai Hospital of Guangdong General Hospital (Foshan, China), and Zhuhai Hospital of Guangdong Provincial People’s Hospital (Zhuhai, China). TBAD was diagnosed according to a multi-detector computed tomography scanning, and patients without complications received TEVAR when the true lumen was severely compressed or when the false lumen was >22 mm in diameter [(10), p. 374–81]. The operation was performed in a cardiac catheterization room with a suitable anatomical structure. According to the pathology of the aorta, the aortic stent diameter generally exceeds the standard by 5–10%. In order to eliminate tears at the proximal entrance, all stent grafts were deployed retrogradely through the percutaneous femoral artery approach. The left subclavian artery was covered when necessary to obtain a 1.5–2-cm proximal landing area. The exclusion criteria were as follows: (1) age <18 years; (2) AD caused by trauma, iatrogenic injury, or intramural hematoma; (3) diagnosis of Marfan syndrome; (4) previous TEVAR or surgery for AD; (5) chronic AD (onset >90 days before treatment) (11); (6) no previous TEVAR interventions; (7) history of malignancy or hematological disease, except anemia; and (8) end-stage kidney disease (Figure 1). The study protocol was approved by the central ethics committee of the Guangdong Provincial People’s Hospital (2017-15), with a waiver for informed consent. This approval by the central ethics committee applied to the other two collaborating hospitals as well.

### Biochemical Assays and Data Collected

Upon admission of the patients, venous blood was collected to determine specific laboratory parameters. RDW was measured using an automated blood cell counter (LH780, Beckman Coulter, Brea, CA, United States), with a normal range of 11–16%. Clinical data were collected through chart review and electronic case reports entered into EpiData software 3.1 (The EpiData Association, Odense, Denmark) and processed using a consistency check on two copies. The estimated glomerular filtration rate (eGFR) in Chinese patients was evaluated using the 4-variable Modification of Diet in Renal Disease (MDRD) equation [(12), p. 2937–44].

### Follow-Up and Endpoints

All patients were followed up by trained researchers *via* telephone interviews or clinic record visits from 9 August 2016 to 30 February 2017. In addition, the full clinical records of readmitted patients and outpatients were reviewed for adverse events. The primary endpoint was long-term mortality, which was defined as death from all causes after being diagnosed with TBAD. In-hospital death, stroke, type I endoleak, and dialysis events were recorded. Type I endoleak is defined as significant when detected during intraoperative control angiography or primary postoperative CTA control (in-hospital) [(13), p. 1022–33, 1033.e15]. Stroke includes ischemic or hemorrhagic stroke, with the clinical diagnosis supported by brain imaging.

### Statistical Analysis

All statistical analyses were performed using SPSS software (version 22.0; SPSS, Inc., Chicago, IL, United States) and R version 3.6.1^1^ (the R Foundation). Mean (±SD) or median (quartile range) values were used to describe continuous variables, which were compared using two independent sample *t*-tests or non-parametric tests based on the data. Categorical variables were reported as absolute values with percentages and compared using the Chi-squared test. Receiver operator characteristic (ROC) curve analysis was performed to evaluate the predictive value of RDW for in-hospital and long-term mortality. In addition, the optimal cut-off values were calculated. The optimal cut-off values for the ROC curves were established using the Youden index. Univariate Cox survival analysis was conducted to assess the risk factors for long-term mortality, indicators with *P*-values less than 0.05 were placed in a multivariate Cox proportional hazard model for further analysis. To visually evaluate the functional relationships between continuous variable and outcomes, the way similar with previous studies explored [(14), p. e007054; (15), p. 4322–9], we used general additive models (GAM) with restricted cubic splines (RCS) to assess the non-linear relationships between RDW and outcomes. Statistical significance was set at *P* < 0.05.

## Results

**FIGURE 2:**
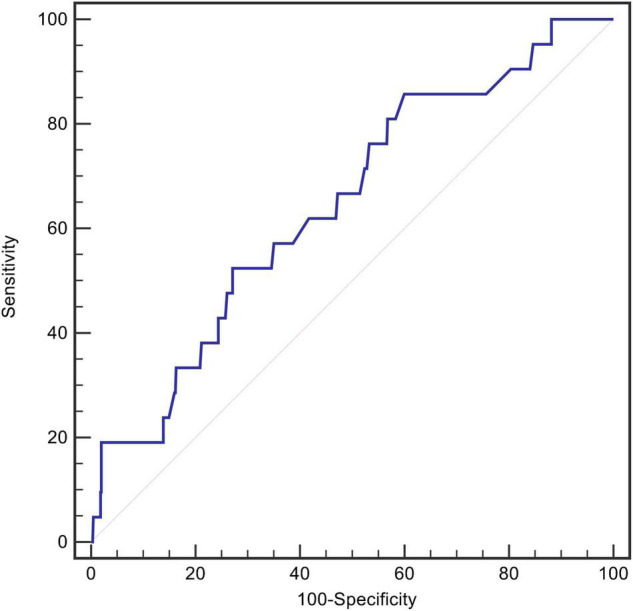
The ROC curves for RDW in predicting in-hospital death.

After screening, data from 678 patients were included in the final analysis. The mean age of the patients was 55 ± 11 years, 86.6% were men, and 21 (3.1%) died during the initial hospitalization. An ROC curve analysis was conducted to determine the predictive value of RDW at admission for in-hospital mortality. The optimal cut-off value was 13.5%, with relatively high sensitivity and specificity (AUC = 0.646, 95% CI, 0.530–0.763, *P* = 0.023, Figure 2).

Patients were classified into two groups according to the threshold value of RDW, with RDW ≤13.5% (*n* = 278) and RDW >13.5% (*n* = 400). At admission, 26.8% of patients developed anemia, with a higher percentage of subjects with a high RDW (33.3 vs. 16.9%, *P* < 0.001). Clinical information was compared between the two groups (Table 1). Hemoglobin and eGFR were lower in the group with a RDW >13.5%, while the serum creatinine was higher.

**TABLE 1:** Clinical characteristics according to RDW cut-off.

Clinical variables	RDW ≤ 13.5% (*n* = 278)	RDW >13.5% (*n* = 400)	*P*
Age (years)	53.7 ± 10.5	55.4 ± 10.9	0.050
Females, *n* (%)	37 (13.3)	54 (13.5)	0.943
Hypertension, *n* (%)	228 (82.0)	347 (86.8)	0.091
Diabetes mellitus, *n* (%)	14 (5.0)	28 (7.0)	0.297
**Type, *n* (%)**			
Acute	223 (80.2)	328 (82.0)	0.558
Sub-acute	55 (19.8)	72 (18.0)	
Hemoglobin (g/L)	130.0 ± 14.5	125.1 ± 19.6	<0.001
Anemia, *n* (%)	47 (16.9)	135 (33.8)	<0.001
CRP (mg/L)	74.0 (28.1, 114.5)	77.4 (26.2,126.8)	0.926
lgDDI	3.11 ± 0.54	3.12 ± 0.53	0.751
Serum creatinine (μmol/L)	97.5 ± 43.1	105.7 ± 50.5	0.024
eGFR (mL/min/1.73 m^2^)	83.8 ± 29.5	78.7 ± 32.6	0.037
LVEF (%)	64.8 ± 5.8	65.0 ± 7.3	0.689
**Artery affected, *n* (%)**			
Celiac axis	84 (32.7)	119 (31.7)	0.801
SMA	48 (18.8)	78 (20.9)	0.506
Right renal artery	54 (21.0)	108 (28.6)	0.032
Left renal artery	68 (26.4)	93 (24.9)	0.673
Pleural effusion, *n* (%)	130 (48.0)	188 (47.5)	0.900
Aortic arch bypass, *n* (%)	45 (16.2)	81 (20.3)	0.181
Stent inserted ≥2, *n* (%)	55 (19.8)	72 (18.0)	0.558
Hospital stay, days	13.0 (9.0, 18.0)	13.0 (10.0,18.0)	0.351
**In-hospital events, *n* (%)**			
Stroke	9 (3.2)	14 (3.5)	0.853
Type I endoleak	20 (7.2)	31 (7.8)	0.787
Dialysis	4 (1.4)	10 (2.5)	0.339
Death	4 (1.4)	17 (4.3)	0.038
Long-term mortality, *n* (%)	15 (6.1)	55 (15.9)	<0.001

*CRP, C-reactive protein; DDI, D-dimer; eGFR, estimated glomerular filtration rate; LVEF, left ventricular ejection fraction; SMA, superior mesenteric artery.*

During hospitalization, 51 (7.5%) patients experienced endovascular leaks after the TEVAR procedure, 23 (3.4%) developed stroke, and 14 (2.1%) required dialysis. There was no statistically significant difference between the patients with an RDW above or below 13.5%. However, the in-hospital mortality was higher in patients with an RDW >13.5% (4.3 vs 1.4%, *P* = 0.038, Table 1).

**FIGURE 3:**
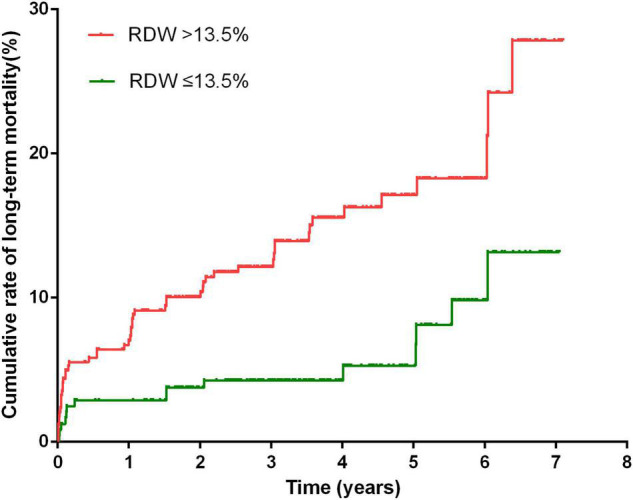
Cumulative rate of long-term mortality in patients with RDW >13.5% and patients with RDW <13.5%.

After a median follow-up of 3.3 years, 70 (10.3%) patients died, and 87 (12.8%) were lost to follow-up. Kaplan–Meier curve analysis showed that patients with an RDW >13.5% had a worse survival than those with a lower RDW (15.9 vs 6.1%, log-rank test = 13.37, *P* < 0.001, Figure 3). The results of the Cox proportional hazard modeling analysis are presented in Table 2. Univariate survival analysis indicated that RDW >13.5% was associated with long-term mortality (HR = 2.78; 95% CI, 1.57–4.92; *P* < 0.001). After the adjustment for these variables, RDW >13.5% remained an independent predictor of long-term mortality (adjusted HR = 2.27; 95% CI, 1.27–4.07; *P* = 0.006).

**TABLE 2:** Univariate and multivariate Cox proportional hazard modeling analysis for long-term mortality.

	Univariate analysis			Multivariate analysis		
Clinical variables	HR	95% CI	*P*	HR	95% CI	*P*
Age	1.05	1.02, 1.07	<0.001	1.05	1.02, 1.07	<0.001
Females	1.82	1.01, 3.27	0.046	2.10	1.14, 3.87	0.017
Hypertension	1.55	0.71, 3.39	0.271			
Diabetes	0.86	0.32, 2.37	0.777			
Acute TBAD	0.93	0.51, 1.69	0.804			
RDW >13.5%	2.78	1.57, 4.92	<0.001	2.27	1.27, 4.07	0.006
Anemia	1.90	1.18, 3.06	0.008	1.22	0.73, 2.02	0.452
CRP	1.00	0.99, 1.00	0.549			
lgDDI	1.68	1.07, 2.65	0.025	1.47	0.93, 2.34	0.100
Serum creatinine	1.00	1.00, 1.01	0.028	1.01	1.00, 1.01	0.019
LVEF	0.97	0.94, 1.01	0.109			
Celiac axis affected	1.23	0.72, 2.10	0.440			
SMA affected	1.73	0.99, 3.05	0.056			
Right renal artery affected	1.42	0.82, 2.46	0.217			
Left renal artery affected	1.08	0.61, 1.91	0.799			
Pleural effusion	1.07	0.67, 1.72	0.768			
Aortic arch bypass	1.28	0.74, 2.21	0.381			
Stent inserted ≥2	1.08	0.58, 2.03	0.801			

*SBP, systolic blood pressure; DBP, diastolic blood pressure; CRP, C-reactive protein; DDI, D-dimer; eGFR, estimated glomerular filtration rate; LVEF, left ventricular ejection fraction; SMA, superior mesenteric artery.*

**FIGURE 4:**
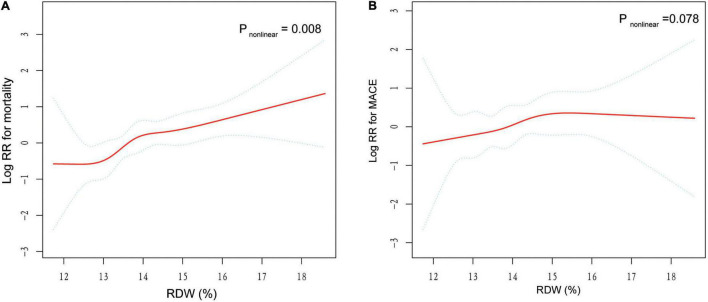
General additive models (GAM) with restricted cubic splines (RCS) demonstrate the relationship between RDW and the risk of mortality and MACE. The resulting figures show the predicted log RR (relative risk) in the *y*-axis and the RDW in the *x*-axis. Log RR can be converted to a relative risk by taking antilog. For example, a log RR of 0 implies the relative risk of 1 (no impact on the probability of prognosis), whereas a log RR of 1 implies the relative risk of 2.71 (i.e., 2.71-fold increase in the probability of MACE or mortality). **(A)** Association between RDW and log RR for mortality. **(B)** Association between RDW and log RR for MACE. The results were adjusted for covariates that statistically significant in multivariate analysis in Table 2, including age, gender, and serum creatinine.

The RCS suggested a non-linear association between RDW and mortality, while this significance was not observed in RDW-MACE relationship (Figure 4). There were increasing relationships between RDW and outcomes. The difference was that, RDW-mortality relation first showed a plateau phase and then increased, while RDW-MACE relation was the opposite. Notably, for RDW-mortality relation (Figure 4A), when RDW (*x*-axis) was 13.5%, the Log RR (relative risk) for mortality (*y*-axis) was approximately 0, suggesting that RR = 1, which implied RDW at this cutoff value had no impact on the probability of mortality. When RDW >13.5%, the Log RR of mortality increased monotonically, reflecting that the increased long-term mortality risk of TBAD patients with the increase of RDW.

## Discussion

To the best of our knowledge, this study is the first to investigate the prognostic value of RDW in patients with TBAD receiving TEVAR. The results showed that RDW could be a predictor of in-hospital mortality. In addition, an RDW >13.5% was independently associated with an increased risk of long-term mortality.

Besides, we found a non-linear RDW-mortality relationship by GAM with RCS, suggesting that there was a threshold effect in the trend of mortality with RDW, and the two-piecewise linear regression models on both sides of the inflection point were statistically significant. As can be seen from the Figure 4A, the RDW of 13.5% might be the inflection point for distinguishing the mortality risk of TBAD patients, corresponding to the RDW cut-off value determined by the ROC curve.

Type B aortic dissection of the descending aorta or arch of the aorta is a life-threatening disease. Open surgical treatment has a high mortality and significant late complications, which have gradually been replaced by TEVAR [(13, 16), p. 1022–33, 1033.e15]. The epidemiological data indicate that the in-hospital mortality rate is 32% for patients with TBAD and treated with surgery, 7% for those treated with TEVAR, and 10% for those treated with medicine only [(17), p. 800–11]. In our study, the long-term mortality rate was 10.3%, which is similar to previous data [(4), p. 407–16]. Despite the decrease in TEVAR mortality, a significant number of patients still die after this intervention. Therefore, there is an urgent need to identify patients with a high mortality risk.

Red blood cell distribution width quantifies the heterogeneity of circulating RBCs and has been used to differentiate the causes of anemia. Subsequently, RDW is a well-established predictor of short- and long-term outcomes in various clinical conditions, including coronary artery disease and infectious diseases, as well as in the general population [(18), p. 515–23; (19), p. 163–8; (20), 123–7]. However, its potential to provide prognostic information for patients with TBAD remains unclear. In this study, we found that increased RDW was associated with in-hospital and long-term mortality after TEVAR for TBAD.

The following features can explain this effect. First, blood could enter the false lumen through the endovascular tear in patients with TBAD, leading to a decrease in circulating blood volume. This phenomenon can be detected by routine blood tests and may present as anemia. In our analysis, 26.8% of the subjects had anemia on admission. A previous study demonstrated a significant increase in RDW in patients with anemia [(8), p. 205–9]. Anemia is a predictor of adverse outcomes in several cardiovascular diseases [(21), p. 610–20; (22), p. 818–27]. However, this result was not the only explanation, as the RDW significance could not be eliminated by including anemia in our multivariate analysis. Second, increased oxidative stress has been found in patients with AD (23). Oxidative stress can directly damage RBCs and decrease their survival, resulting in increased anisocytosis (24, 25) and increased RDW. Selenium was negatively correlated with RDW as a component of the antioxidant defense system (26). A higher RDW may reflect severe oxidative stress, leading to adverse outcomes. Third, stress on the endoplasmic reticulum might occur, altering the regulation of apoptosis and inflammation. Excessive apoptosis could subsequently promote inflammation and degeneration of the vascular wall, which is an important mechanism for the formation and progression of AD (27). In response to this inflammatory process, pro-inflammatory cytokines are released, resulting in an increase in RDW [(28), p. 1011–23]. Finally, shear stress may be another factor leading to a high RDW and adverse outcomes. Taguchi et al. found that a high shear stress could induce intimal tears, which can progress to overt AD [(29), p. 78–82]. In addition, increased shear stress was associated with the development of retrograde aortic type A dissection, which is a severe complication of TBAD [(30), p. 324–330]. Shear stress can also impair RBC deformation and precipitate hemolysis, promoting increased RDW [(31), p. 1017–25].

The current study found that an increase in RDW levels upon admission is independently associated with an increase in long-term mortality in patients with TBAD. As an easily available indicator in routine blood testing, RDW can be used as an important factor in prognostic risk stratification, selecting high-risk patients, guiding treatment strategies, and reducing the risk of all-cause death in patients with TBAD. In view of the common prognostic effect of high RDW in several cardiovascular and infectious diseases, a high RDW is likely to represent secondary damage related to inflammatory responses, and oxidative stress. Monitoring changes in RDW may reflect the effects of controlling cardiovascular risk factors. It may be possible to base future follow-up plans on patients’ RDW and improve the long-term survival of AD patients, since many patients with TBAD may not strictly follow the annual follow-up recommendation.

### Study Limitation

This study had several limitations. First, due to its retrospective design, confounding factors may have affected the results. However, a multivariate analysis was performed to reduce these. Second, the predictive value of RDW was not compared with that of other inflammatory markers. Third, the RDW was not detected dynamically. Therefore, it is not clear whether a continuous measurement of the dynamic changes in RDW rather than the measurement of the baseline RDW would have a better predictive value for the outcomes of the patient. Finally, the lost to follow-up rate (12.8%) in the present study was relatively high and could lead to selection bias.

## Conclusion

As an inexpensive and routinely measured RBC parameter, RDW has been proven to be a useful prognostic marker in patients with TBAD receiving TEVAR. Increased RDW was significantly associated with in-hospital and long-term mortality. This relationship should be investigated in prospective studies with larger sample sizes.

## Data Availability Statement

The original contributions presented in the study are included in the article/supplementary material, further inquiries can be directed to the corresponding authors.

## Ethics Statement

Ethical review and approval was not required for the study on human participants in accordance with the local legislation and institutional requirements. Written informed consent for participation was not required for this study in accordance with the national legislation and the institutional requirements.

## Author Contributions

QG, YL, and CJ contributed to the conception and design of the study. CJ, AL, LH, and QL contributed to the acquisition, analysis, and interpretation of data. CJ and AL drafted the manuscript. QG and YL critically revised the manuscript. All authors gave final approval and agreed to be accountable for all aspects of work ensuring integrity and accuracy.

## Conflict of Interest

The authors declare that the research was conducted in the absence of any commercial or financial relationships that could be construed as a potential conflict of interest.

## Publisher’s Note

All claims expressed in this article are solely those of the authors and do not necessarily represent those of their affiliated organizations, or those of the publisher, the editors and the reviewers. Any product that may be evaluated in this article, or claim that may be made by its manufacturer, is not guaranteed or endorsed by the publisher.

## Footnotes

1
http://www.R-project.org
